# The selfless sacrifice of doctor Junqiao Zhang: leaving a lasting legacy in China- Africa health cooperation

**DOI:** 10.1186/s41256-025-00446-6

**Published:** 2025-08-29

**Authors:** Bingqing Xi, Hao Li

**Affiliations:** School of Public Health/Global Health Institute, Wuhan University, Wuhan, 430071 China

On the afternoon of 15 June 2025, a sudden drowning accident on the coast of Dar es Salaam, Tanzania, ended in tragedy. Doctor Junqiao Zhang—head of the 27th China Medical Team (CMT) from Shandong Province of China to Tanzania—lost his life after exhausting himself while rescuing a drowning local civilian.

China's medical aid to Tanzania dates back to 1964, with two teams deployed over the years. One team, dispatched by Jiangsu Province starting in August 1964, has been stationed in Zanzibar [1]. As of November 2024, a total of 34 batches involving 847 health professionals have been sent to Zanzibar [1]. The other team, sent by Shandong Province from March 1968, has been assigned to Tanzania mainland [2]. By February 2025, 27 batches comprising 1,084 health professionals had been deployed [2]. These CMT members have made significant contributions to fostering China-Tanzania friendship and served as a vital bridge and bond between the two nations. Doctor Junqiao Zhang is among them. As the leader of the 27th CMT, Doctor Junqiao Zhang carried forward a commitment spanning more than half a century.

In January 2024, Doctor Zhang arrived at Muhimbili National Hospital in Tanzania with 10 medical team members from Shandong Province, China for a two-year medical aid assignment [2]. In the operating rooms of the hospital, he had worked as both a skilled anesthesiologist and a patient mentor. Confronting a severe shortage of local anesthesia professionals in Tanzania, he introduced techniques such as ultrasound-guided nerve block and mentored Tanzanian doctors one-on-one. At an orphanage in Dar es Salaam, he has been affectionately known as the "Chinese Doctor" by over 80 orphans, providing free medical consultations, distributing medicine, and teaching hygiene and protective measures [3]. At construction sites of Chinese enterprises, he traveled between three projects three times, spreading the knowledge of Cardiopulmonary Resuscitation and Automated External Defibrillator to every employee. As a representative of a Chinese enterprise remarked, "Even after teaching Cardiopulmonary Resuscitation hundreds of times, the passion in his eyes never faded [4]."

"We must train people and leave things behind"—this was his unwavering vow in the face of the shortage of medical resources in Tanzania [3]. Muhimbili National Hospital, the country's top medical institution, had long struggled with a shortage of anesthesiologists, therefore many complex surgeries have to be postponed. After Doctor Zhang's arrival, he not only performed numerous high-risk anesthesia procedures but also established a systematic training program. He organized specialized anesthesia training sessions for local medical staff and students, teaching them techniques such as visualized airway management, ultrasound-guided peripheral nerve block, and combined general and high epidural anesthesia to reduce anesthesia risks. Faced with the outdated anesthesia equipment, he also facilitated the donation of a visual laryngoscope worth over 100,000 RMB to the hospital through Shandong Second Medical University [5].

His ultrasound-guided peripheral nerve block training was hailed by Tanzanian experts as "urgently needed cutting-edge medical knowledge for Tanzanian medical staff and students" [3]. His simple wish of "training more medical staff and passing on knowledge" is gradually becoming a reality [4]. Today, the intubation techniques he taught continue to save lives in operating rooms, and the training courses he designed are still nurturing local medical talents [3]. As head of the hospital's anesthesia department noted, Doctor Zhang not only brought technology but also built an emotional bridge connecting doctors from both countries [3].

On 20 June 2025, local time, over 300 Tanzanians, medical staff, and members of the Chinese community gathered at Muhimbili National Hospital to bid farewell to Doctor Zhang [3]. Tanzania’s Ministry of Health expressed on social media that Doctor Zhang had been a devoted and esteemed anesthesiologist, whose demise represented a significant loss not only to the medical profession but also to the numerous individuals who had benefited from his compassionate care, professional expertise, and steadfast commitment to patient welfare [4]. Doctor Zhang's last post on social media read: "Serving the people knows no borders." He lived up to his words with his life. He is just one of many such cases from CMT members.

The CMT program, first started in 1963 in Algeria, with doctors sent from Hubei Province, has undergone sixty two years of practice, which not only met local medical needs and improved the quality of care, but also strengthened the capacity building of local medical teams, exerting long-term and far-reaching impacts on the healthcare systems in the aided regions [6]. Up till 29 August 2024, China has sent 25,000 medical professionals to 48 African countries while more than 50 CMT members have lost their lives for various reasons, such as diseases, conflicts, occupational injuries, and accidental incidents [7, 8]. Their stories are deeply moving. For generations, these stories have been shared across the African continent. However, their deeds are yet to receive sufficient coverage in international journals, particularly those based in low and middle income countries. Therefore, our journal of Global Health Research and Policy would like to take the initiative to tell their stories now and in the future. We hope that, by telling more stories of their overseas medical work and sacrifice spirit, we can raise public awareness and understanding of this group and thereby advance China-Africa medical cooperation. By doing so, the China-Africa friendship and solidarity are expected to flourish in the common cause of safeguarding lives and to light the path forward for more people, contributing to building a global community of health for all.

## Data Availability

Not applicable.

## Abbreviation

CMT: China medical team
